# No free lunch for avoiding clustering vulnerabilities in distributed systems

**DOI:** 10.1038/s41598-024-63278-3

**Published:** 2024-06-04

**Authors:** Pheerawich Chitnelawong, Andrei A. Klishin, Norman Mackay, David J. Singer, Greg van Anders

**Affiliations:** 1 Department of Physics, Engineering Physics, and Astronomy, Queen’s University, Kingston, ON K7L 3N6 Canada; 2 Department of Mechanical Engineering, University of Washington, Seattle, WA 98195 USA; 3 AI Institute in Dynamic Systems, University of Washington, Seattle, WA 98195 USA; 4 Department of Naval Architecture Marine Engineering, University of Michigan, Ann Arbor, MI 48109 USA

**Keywords:** Statistical physics, Complex networks, Engineering

## Abstract

Emergent design failures are ubiquitous in complex systems, and often arise when system elements cluster. Approaches to systematically reduce clustering could improve a design’s resilience, but reducing clustering is difficult if it is driven by collective interactions among design elements. Here, we use techniques from statistical physics to identify mechanisms by which spatial clusters of design elements emerge in complex systems modelled by heterogeneous networks. We find that, in addition to naive, attraction-driven clustering, heterogeneous networks can exhibit emergent, repulsion-driven clustering. We draw quantitative connections between our results on a model system in naval engineering to entropy-driven phenomena in nanoscale self-assembly, and give a general argument that the clustering phenomena we observe should arise in many distributed systems. We identify circumstances under which generic design problems will exhibit trade-offs between clustering and uncertainty in design objectives, and we present a framework to identify and quantify trade-offs to manage clustering vulnerabilities.

## Introduction

A key challenge in the design of complex, large-scale systems is managing emergent vulnerabilities^1–5^, especially those driven by clustering. Clustering instabilities are common in systems that are comprised of a large number of mutually dependent units. Early applications of ideas from network theory to the large-scale design of distributed systems delivered fundamental insight into the way that network topology acts as a driver of cluster-driven vulnerabilities. The analysis of clustering vulnerabilities using network-theoretic approaches provides considerable value^6–9^ in settings where details of the physical realization of the network topology are negligible.

Network-theoretic techniques capture clustering effects in some very important cases^6–9^, yet because purely network-theoretic analyses abstract out physical realizations, they fail to capture clustering vulnerabilities that arise in many real-world systems through an interplay of network topology and spatial proximity. Spatial proximity becomes relevant in any situation in which the networks are realized with physical edges that are characterized by length scales that are comparable to important operating considerations. A simple example of this could occur in transportation systems that connect locations separated by distances on the typical scale of major storm systems. Examples of systems where network and spatial effects combine to drive clustering vulnerabilities include hot spot leakage in microprocessors^10,11^, congestion in airline networks^12,13^, and high outfit density in naval engineering^14,15^. Work has identified key aspects of spatial proximity in lattice networks^16^, but the effects of clustering vulnerabilities on the topologically and spatially heterogeneous networks that describe many real-world systems are poorly understood.

Understanding and managing clustering vulnerabilities in settings with topological and spatial heterogeneity are crucial because they involve pernicious local/global interactions. Clustering vulnerabilities, by definition, arise locally, but their performance effects are frequently global. For example, mitigating isolated hot spots in microprocessors frequently involves throttling the performance of the entire device^17–19^. Similarly, damage to a single shipboard system can cause co-located systems to fail, inducing failures that cascade through the ship^20–23^. The existence of important local/global interactions raises the stakes for mitigating clustering vulnerabilities.

A further complication that arises in many settings is the insufficiency of single-solution optimization approaches. The insufficiency of “one-and-done” design approaches that produce a single optimal design is most acute in domains that rely on yet-to-be-proven technologies.

The problem with one-and-done approaches to design can be illustrated with the example of naval architecture. To maintain operational advantage, naval vessels must be launched with technologies that represent the state-of-the-art at the time of launch. Moreover, after they go into operation, they must permit retrofitting as technologies advance. Because of long development and manufacturing times, there are typically significant advances in the state of the art for key technologies over development and deployment timescales. This timescale mismatch means that the design process input contains extrapolations that estimate key performance outcomes. The intrinsically extrapolative structure of the design process implies that early-stage design processes cannot rely on design frameworks like optimization algorithms that aim to produce a single optimal solution. In any given design problem, the chances that all extrapolations will work out precisely is vanishingly small. Moreover, subsequent retrofits in operation compound the problem. Although this issue is particularly salient in naval architecture, similar challenges plague other domains. For example, the emergence of integrated circuits for general purpose computing means that the compute loads that chips will support in operation cannot be precisely known during design. Uncertainty in design and operation raises the challenge of not merely needing to manage clustering issues in a single instance. Instead, uncertainty requires managing clustering in the face of a future that is certain to differ from present knowledge.

Techniques to manage future uncertainty also make it necessary to analyze designs statistically. Because of the inevitability of uncertainty in many design problems, having a variety of ways to rework or alter implementations provides a means to hedge against uncertainty. Statistical analyses provide a means to this end. However, enacting a statistical approach necessitates introducing some distribution of possibilities that does not introduce undue bias in the analysis.

If clustering vulnerabilities engender such significant and pernicious risk, how can they be such ubiquitous outcomes of ostensibly rational design processes? Topological drivers of clustering vulnerabilities can be driven by the functional relationships among design elements, e.g., elements that supply or consume energy^14,24^. Spatial drivers of clustering vulnerabilities arise in complex system design^2,3,6,25–27^, because in systems comprised of a large number of functional units, units are arranged to minimize physical connection distances. Distance-minimization drivers can be economic, e.g., minimizing material cost^28,29^, or physical, e.g., minimizing energy loss or latency^7,30–32^, or a combination of economic and physical^33,34^. Regardless of the driver, connection-distance minimizing arrangements of design elements induce spatial grouping, and that grouping produces clustering vulnerabilities^35,36^.

Because clustering vulnerabilities are driven by an interplay of logical and physical requirements of the system,^37^ mitigating vulnerabilities requires attending both to networks of functional connections and to the physical realizations of those connections in space. Moreover, because topological drivers of clustering vulnerabilities are the product of primary functional relationships, there may be little latitude for mitigation without sacrificing inviolable functional requirements. The narrow latitude for solely topological mitigation raises a greater need for approaches that attend to the key role that spatial realization plays in generating clustering vulnerabilities. Moreover, new methods need to do this in manners that do not artificially proscribe classes of potential outcomes.

Since clustering vulnerabilities arise generically in the optimization of systems with complex inter-dependencies, there are two obvious strategies to mitigate such vulnerabilities. One obvious mitigation strategy is to make ad hoc modifications to the optimization criteria to counteract the clustering that produces the vulnerability. However, vulnerabilities that are most difficult to manage are emergent ones, and they are most likely to arise in, e.g., complex systems described by large networks of connected elements^1^. Because emergent vulnerabilities occur unpredictably, actions to mitigate one vulnerability may induce the emergence of others, which could be more numerous or severe than the original. If modifying optimization criteria ad hoc to mitigate one vulnerability can drive the emergence of others, a second alternative strategy could be to look beyond strictly optimal solutions in a systematic way. In situations where economic considerations factor among optimization criteria considering non-optimal solutions is, in a colloquial sense, buying a way out of the problem. However, employing expensive, sub-optimal solutions could be a worthwhile sacrifice if the vulnerabilities induced by clustering are severe, and if sub-optimal solutions reliably eliminate clustering.

Here, we employ Pareto-Laplace filters^38^ to systematically examine design solutions away from the strictly optimal limit. The Pareto-Laplace filter allows for constructing sets of putative design solutions according to some design objectives that uses information theory to make minimal assumptions about other design features. We show that a complex interplay between how design elements are physically arranged and how the elements are functionally connected defeats simple strategies to mitigate cluster-driven vulnerabilities. We illustrate this with examples computed for a model from naval architecture. The naval architecture examples suggest the existence of a more general underlying mechanism that occurs whenever there exists a multiplicity of possible choices of connection routes for each given placement of functional elements in a fixed spatial region. We used the multiplicity observation to draw an analogy to contrasts between configurational and conformational entropy in physical systems to construct an argument that routing multiplicity is the dominant driver of element placement when connection-distance minimization is relaxed in generic settings. Colloquially, one might describe this as akin to “the tail wagging the dog.”

Altogether, we find that the analogue of a configurational/conformational entropy trade off has two key implications. One implication is that, under minimal, information-theoretic assumptions, the complex interplay between topological connectivity and spatial arrangement gives rise to unexpected, emergent clustering when requirements for distance minimization are severely relaxed. Another implication is that if distance-minimization requirements are only partially relaxed, declustering can occur, but that declustering coincides with maximal variability in the primary design objective. Our results indicate that there is no simple, one-size-fits-all approach to managing clustering vulnerabilities. However, although there is “no free lunch” for managing clustering vulnerabilities, the approach we present gives a framework to manage them in a context-dependent, case-by-case manner.

## Results

### Clustering in a model system

To motivate a general argument on clustering vulnerabilities, it is instructive to first understand how they arise in a specific example. We use a system from naval architecture that describes arranging the power system of a naval vessel inside a ship hull^14^. The model describes the placement of elements of the power system and their interconnections, with a cost associated with connection length (see Methods). This model has three features that are exemplary of other contexts: (1) optimizing arrangements for short connection distances drives system elements to cluster in space^39^; (2) because the network is comprised of elements that are power sources and power sinks, the connectivity of the network is heterogeneous; i.e., it has both high- and low-connectivity elements; (3) the network is bipartite with two group of elements: power sources and power loads.

We define a microstate of the system as a candidate arrangement of all elements in the system, including the positions of all of the nodes and the routes of connections between the nodes. Hence, the solution space is comprised of all possible arrangements in space of the design components in the system. We study this model by generating candidate arrangements controlled by a parameter *T* (see Methods) that serves as a tolerance for generating non-minimal routing distances, and is mathematically equivalent to temperature (see Methods). $T=0$ indicates no tolerance for non-minimal routing distance and $T\rightarrow \infty$ indicates unlimited tolerance for non-minimal routing distance. Results below parametrize *T* relative to a crossover value $T_c$ that we determine by comparing classes of model results (see Methods).

**Figure 1:**
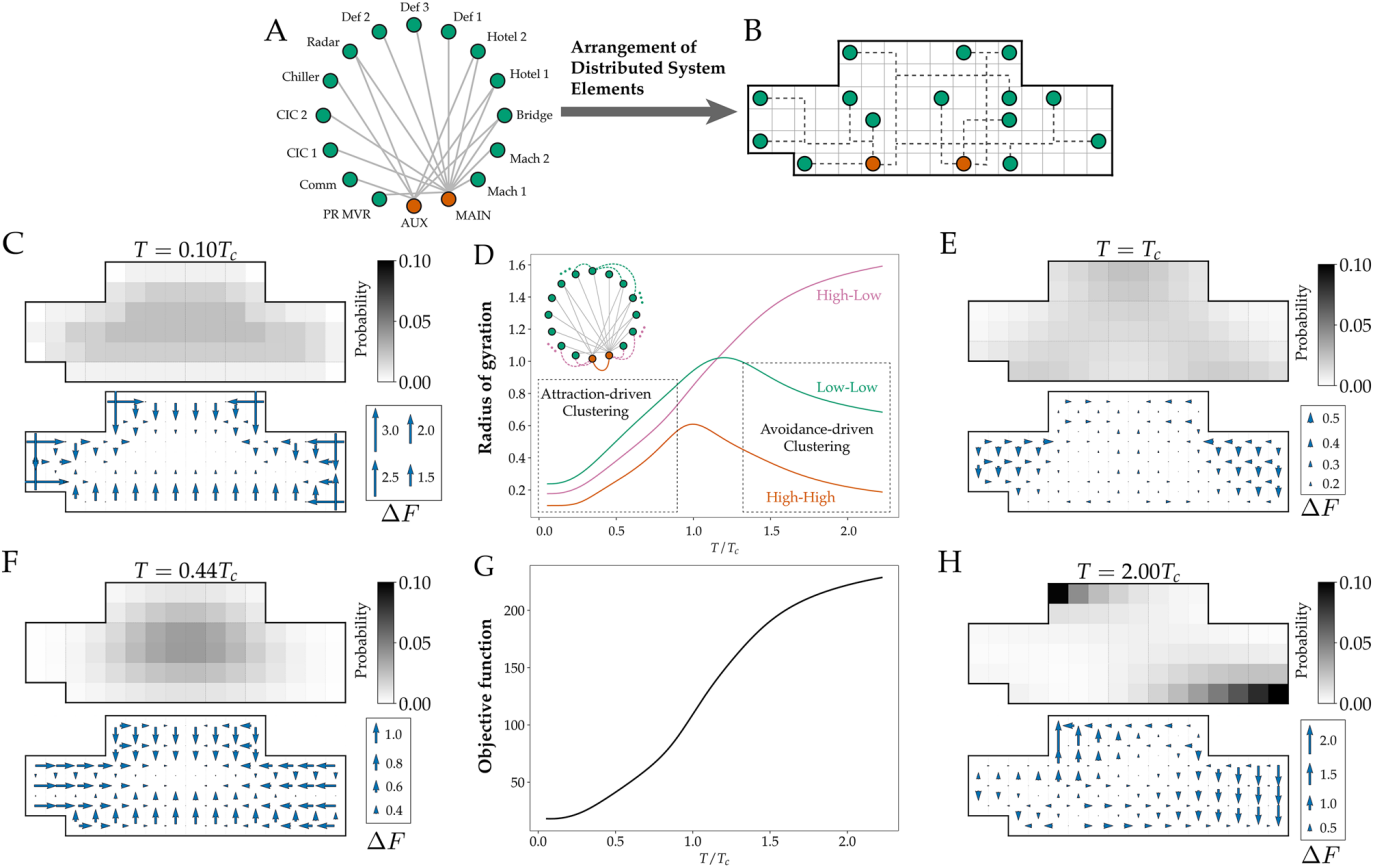
Avoidance-driven clustering emerges in non-minimal distance routing. (**A**) Illustrates design-element connectivity of a shipboard power systems. (**B**) Illustrates a hypothetical arrangement. (**D**) Plots radius of gyration ($R_g$) versus T that quantifies correlations between elements paired by their degree of connectivity, low-low, high-high, and high-low. Low $R_g$ at low T, where designs are dominated by minimal routing distance are clustered by attraction. However, low $R_g$ at high T for functionally disconnected, low-low and high-high connectivity pairs that coincides with high $R_g$ for functionally connected high-low pairs indicates repulsion driven clustering. The inset network diagram shows example element pairs that $R_g$ is averaged over. (**G**) Plots the value of the objective function as internal energy of the system versus temperature. (**C**, **E**, **F**, **H**) Show element localization in the ship hull. Low T clustering (**C** and **F**) is expected since the objective prioritizes routing cost, and the distribution is a single dense region, that is central, and excludes the boundary, all consistent with attraction-driven clustering. High T clustering (**H**), however, is multi-modal and peripheral. Moderate T (**E**) corresponds to local peaks in $R_g$ in (**D**) and is distributed throughout the hull which indicates de-clustering.

Figure 1 reports clustering behaviours in our model system. We use two types of measure: global and local. For the global measure, we borrow from polymer physics and measure global clustering via the radius of gyration, $R_g$^40^, a root-mean-squared distance between a set of objects (see Methods). The radius of gyration reports average ‘pair-wise’ (two-point) correlation between all pairs, regardless of the position of the design element in the ship. The local measure reports an average one-point correlation over all design elements over different locations of the ship hull. We report both global measures of clustering (panel D) and local measures in the ship hull (panels C, E, F, H) at a range of temperature (tolerances for non-minimal route length).

#### Local measures show emergent clusters are peripheral

To understand the origin of emergent clustering, we analyzed the local distribution of power system elements throughout the ship hull. We first establish a baseline for comparison by computing element distributions at low *T*, where we expect conventional attraction-driven clustering.

Figure 1C shows the distribution of power system elements in arrangements driven by attraction-driven clustering. The global measure of clustering at low *T* in Fig. 1D indicated that the system elements form a cluster with small $R_g$. The local distribution in Fig. 1C shows that the global clustering coincides with arrangements with a near-uniform distribution throughout the ship hull. Note that the exception to uniformity is the depleted region near the boundary. This behaviour is analogous to the behaviour of polymer solutions confined, for example, within a tube with repulsive walls where the conformational entropy, i.e., internal rearrangements, of the polymer reduce the density of the solution near the walls^41^. As further confirmation, increased but still relatively low *T* (from $0.2 T_c$ to $0.4 T_c$) produces clusters with increased $R_g$, which lead to element distributions with a wider depleted boundary layer. These distributions share three features: a single dense region, central distribution, and boundary exclusion.

#### Global measures show emergent clustering of non-connected elements

At high *T*, distributions show that they are multi-modal, not centred, and do not exclude the boundary. Figure 1H shows that high *T* ($T = 2T_c$) generates element distributions that exclude the central region; instead they localize on the boundary in two distinct regions. The existence of these two regions accords with the global $R_g$ clustering measure: Fig. 1D showed that functionally disconnected elements that had similar degrees of connectivity (low or high) formed clusters, whereas functionally connected elements had large $R_g$. The element distribution in Fig. 1H suggests the differing clustering of functionally disconnected elements (low-low and high-high, which cluster) and functionally connected elements (high-low, which spread) arise because distinct regions of the element distributions correspond to elements with distinct degrees of connectivity.

We note that the underlying form of the power system as a set of objects “tethered” to one another by functional connections is reminiscent of polymer systems. As expected, at low *T* we find that power system elements are tightly clustered, regardless of their degree of network connectivity, as indicated by low gyration radii.

More surprisingly, however, we find that clustering re-emerges when *T* is high. Figure 1D shows that although $R_g$ approaches the size of the space for directly connected power system elements (all of which are high-low connectivity pairs), $R_g$ is small for unconnected power system elements (low-low and high-high connectivity pairs). This form of clustering is striking for two reasons: because clustering involves subsets of the elements, and because the elements that cluster together are ones that don’t have direct functional connections.

#### Emergent, peripheral clusters segregate by degree of connectivity

**Figure 2:**
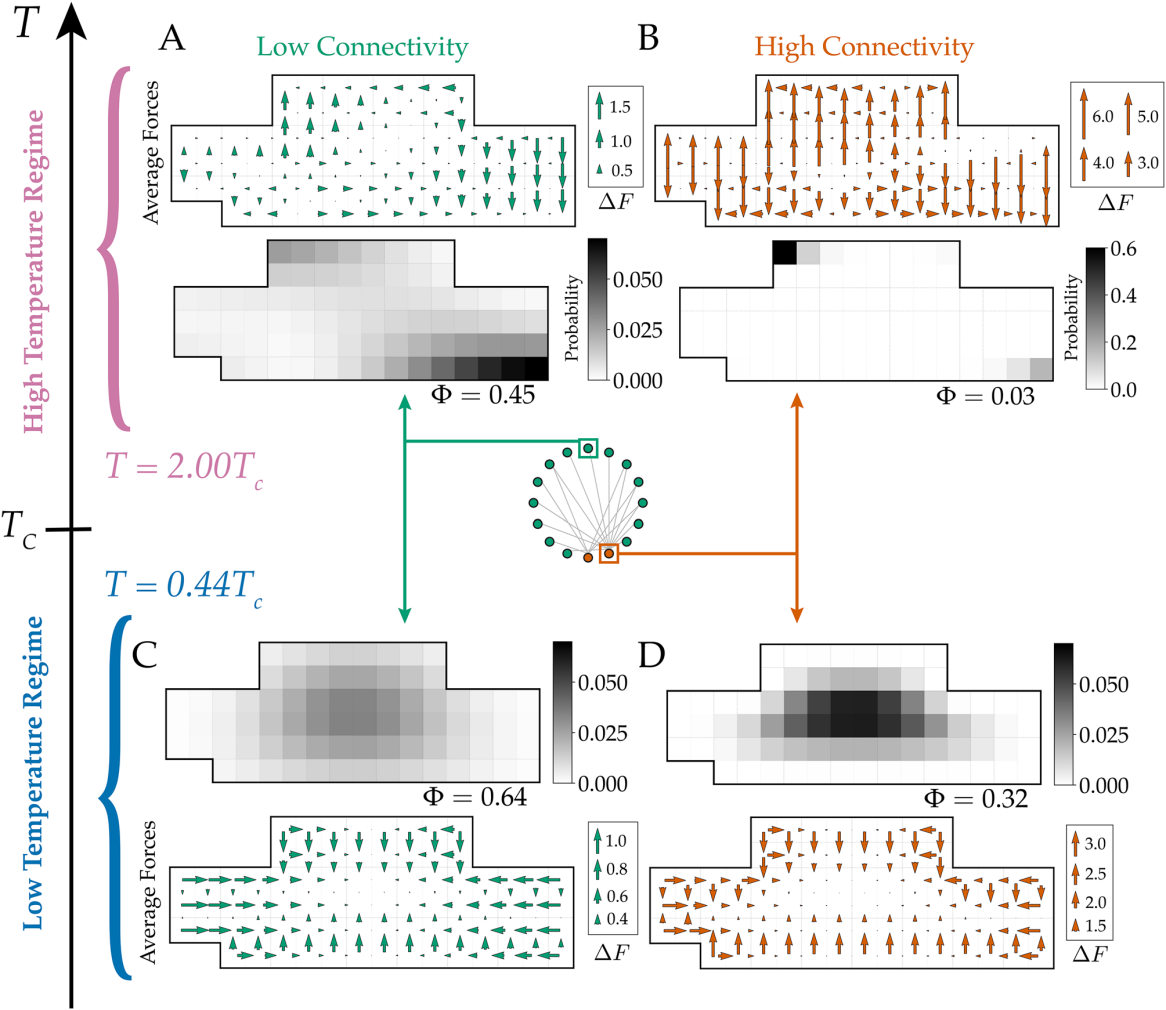
Emergent clustering segregates design elements by degree of connectivity. Panels A-D show probability distributions and effective forces for representative design elements that have low- (AC) or high- (BD) degrees of connectivity. Avoidance-driven clustering at high *T* in low-connectivity elements (**A**) and high-connectivity elements (**B**) display distinct behavior. Low connectivity elements adhere to the bottom right of the hull, and high-connectivity elements localize near the top left. Effective forces (quiver plots) are larger for high-connectivity elements. Attraction-driven clustering at low *T* for low-connectivity (**C**) and high-connectivity (**D**) elements shows high-connectivity elements are more localized, which is consistent with the force measurements.

To determine whether cluster separation occurs because elements separate by degree of connectivity, we separately analyze the distribution of representative high-connectivity and low-connectivity power system elements in Fig. 2. Computed distributions indicate that the two concentration areas in the connectivity-agnostic element distribution in Fig. 1H can be associated with either high- or low- connectivity elements. Panels A and B in Fig. 2 indicate that emergent clusters segregate elements by their degree of connectivity.

For comparison, we computed distributions for the same elements at low *T*. Panels C and D in Fig. 2 suggest that, while there is a considerable overlap between distributions for high- and low-connectivity elements, high-connectivity distributions are more concentrated suggesting a “core-shell” form of spatial organization.

#### Routing multiplicity drives emergent clustering

The existence of clustering at low *T* is unsurprising, however the re-emergence of clustering when the preference for non-minimal routing is relaxed is unexpected. The fact that clusters form in separate, segregated, peripheral groupings violates the intuition that power system elements should de-localize if the drive for minimal routing is relaxed.

To understand the origin of the unexpected emergent clustering, it is instructive to extend the analogy with conventional physical systems. The generating function for arrangements (see Methods) can be decomposed into three sets of contributions: the length-dependent cost of routing connections between power system elements, the multiplicity of arrangements of power system elements with fixed route lengths, and the multiplicity of the routing paths for a fixed element arrangement and route lengths. These factors are analogous to line tension, configurational, and conformational entropy, respectively, in physical systems. The identification of these physical analogues gives a direction for further analysis.

**Figure 3:**
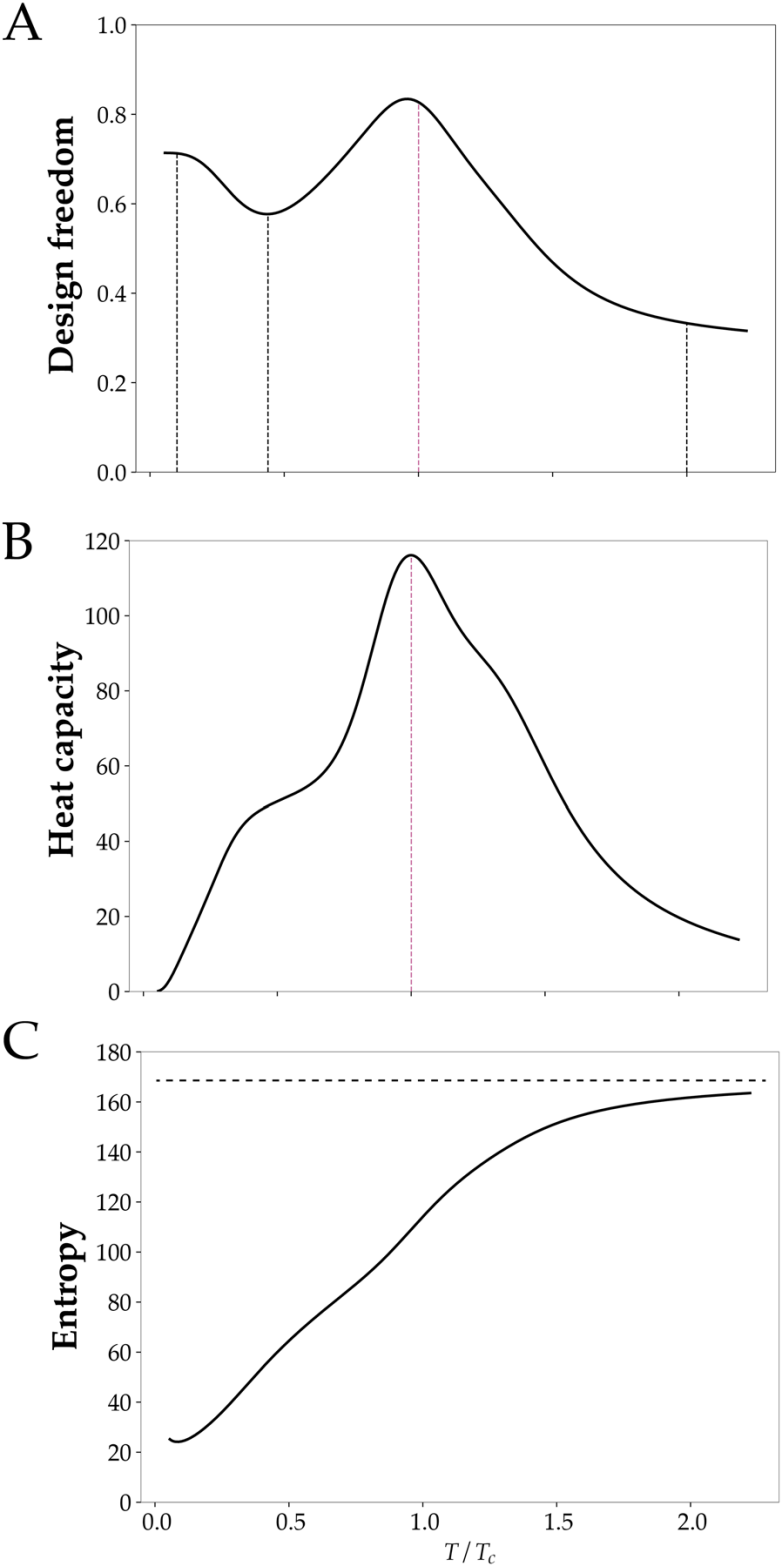
Declustering coincides with high-variability in the design objective. **(A)** design freedom (a measure the freedom to place design elements, see Methods) vs *T* peaks at intermediate *T*, indicating declustering. **(B)**, heat capacity vs *T*, indicates that declustering coincides with a peak in heat capacity. However, because heat capacity measures fluctuations in routing distance (see Methods), declustering coincides with maximal uncertainty in the primary design objective. **(C)**, total system entropy vs *T* increases monotonically, as expected, indicating that decreasing design freedom at large *T* occurs because routing multiplicity increases at the expense of unit placement multiplicity.

The analogy between power system element arrangement multiplicity and configurational entropy suggests quantifying this contribution in terms of the spatial spread of the element distribution. In other contexts, existence area (see Methods) is used to measure inhomogeneity in distributions that arises in localization^42^. Here, we use the same mathematical form to characterize the arrangement multiplicity of power system elements. Figure 3A shows this form of design freedom, which is a proxy for configurational entropy, as a function of *T*. Decreases in design freedom at low *T* and at high *T* are counter-intuitive because they indicate a loss of configurational entropy.

In thermodynamic systems, entropy conventionally increases monotonically with temperature, which is typically expressed in terms of a strictly positive heat capacity, $C_V$. Figure 3B,C show total system heat capacity and entropy as a function of *T*. Figure 3C shows that entropy is an increasing function of *T* as expected, and Fig. 3B shows that the heat capacity is strictly positive. These results indicate that the reductions in design freedom with increasing *T* still coincide with increasing total entropy, but that this total entropy increase occurs because the conformational entropy associated with the existence of multiple routing paths between a fixed arrangement of elements overwhelms the configurational entropy associated with the multiplicity of element arrangement.

**Figure 4:**
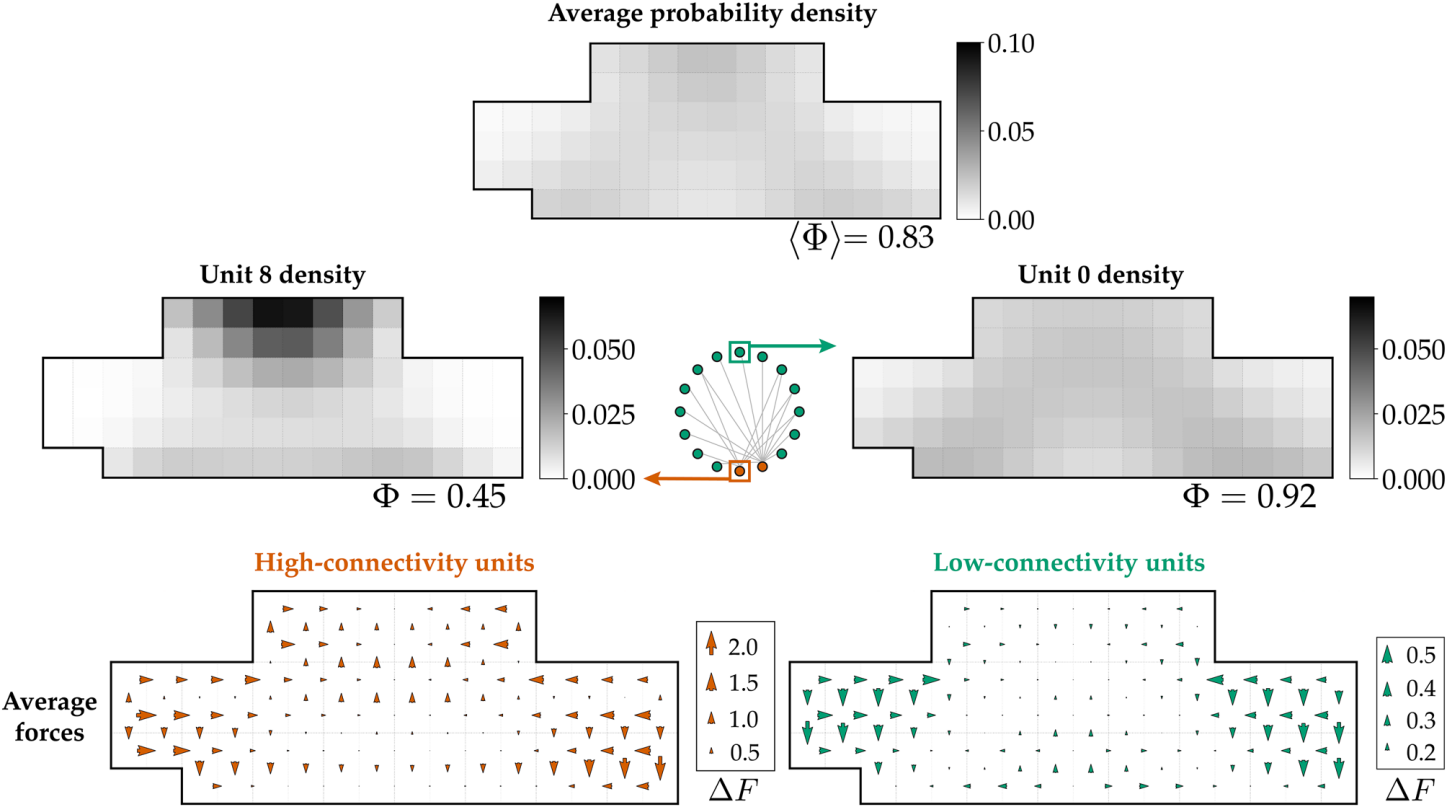
High variability configurations segregate by connectivity. High variability configurations at intermediate *T* ($T=T_c$) have distributions that differ by connectivity. High-connectivity elements (left panels) localize more strongly than low-connectivity elements (right panels).

Taken together, five factors all suggest emergent clusters that are separate, segregated, and peripheral are driven by the generation of arrangements that are dominated by the multiplicity of routing paths for a fixed arrangement of power system elements: (1) the global clustering $R_g$ (Fig. 1D); local element distributions, both (2) agnostic of connectivity (Fig. 1H) and for representative (3) low- (Fig. 2A) and (4) high-connectivity (Fig. 2B) elements, and (5) contrasting design freedom and entropy measures.

#### Declustering coincides with design objective variability

The above analysis showed that the unexpected re-emergence of clustering at high *T* was driven by entropic effects. However, this analysis also revealed a peak in the heat capacity in Fig. 3B at intermediate *T*, and this raises the possibility of a different scenario to avoid clustering.

In systems of macroscopic numbers of atoms, sharp divergences in heat capacity signal a phase transition at a corresponding critical temperature. And, importantly, conventional thermodynamic systems at a critical point typically develop fractal behaviour, with spatial organization at many different scales^43^. Multi-scale organization is a possible “out” to the clustering problem, and could be achievable, not at high *T* where instead we observed re-emergent clustering, but at intermediate *T*.

The present system has a finite number of elements so the heat capacity cannot exhibit a sharp divergence. However, despite the lack of a sharp divergence in heat capacity in the present system, quasi scale-invariant behaviour is possible. To investigate this we carried out global and local measures of clustering at $T_c$. Figure 1E shows that indeed power system elements distribute in both central and peripheral locations. This spread-out distribution approximately coincides with maximal $R_g$ for non functionally-connected elements (Fig. 1D), and a peak in design freedom (Fig. 3A).

Furthermore, segregation by connectivity for high- and low- connectivity elements is also found at intermediate temperature as shown in Fig. 4. However, the clustering behaviour is significantly mitigated with the average design freedom of 0.83 at $T=T_c$. This result emphasizes that connectivity-dependent localization is an inherent feature of a heterogeneous network that persists across all temperatures.

Together, the results indicate that in the vicinity of $T_c$ there is a significant decrease in clustering. However, this decreased clustering comes at a price. Thermodynamic heat capacity serves as a measure of the size of energy fluctuations across a set of states^44^. Comparing the present system with thermodynamic systems, the role of energy is taken by the total routing cost, and so high heat capacity implies high routing cost uncertainty. This routing cost uncertainty, however, implies high variability in the main design objective. This means that in the present system, declustering coincides with high design objective variability.

## Discussion

### Clustering by repulsion-driven attraction in general

The clustering behaviour we observed in the naval architecture model arose from an interplay between the objective for minimal-length routes and two multiplicities: arranging power system elements and arranging the connections between them. These two forms of multiplicity are directly analogous to configurational and conformational entropy that drive clustering and arrangement in other systems^45^. A well-studied example of this is the self-assembly of tethered nanoparticles^46^. In these nanoscale systems, a subtle interplay between the entropy of the nanoparticle configurations and the conformations of polymer tethers drives complex, emergent organization, including the clustering of non-attracting objects^47^. The existence of a nanoscale analogue of the behaviour we observed in our naval architecture model strongly suggests the model behaviours we observed signal a manifestation of a more general phenomenon.

To see this, consider a generalization of the model arrangement problem we analyzed above. The general model is a system of *N* elements to be placed at some positions $\vec{x}_i$ in a domain *D*, where the subscript labels the element, and the vector components are coordinates of the position of that element. It is most concrete to think of the coordinates describing positions in physical space, however they could also describe positions in the space of element specifications (e.g., power consumption). We consider a situation in which some of the objects are functionally connected to one another, and some are not, which we encode in an adjacency matrix $A_{ij}$, which is one if elements *i* and *j* are functionally connected and zero if they are not.

Understanding whether clustering occurs in arranging design elements requires a means to generate arrangements systematically. Systematically generating candidate solutions to a problem that makes minimal assumptions about the form of the solutions is governed by the theory of information^48^. Information theory shows^39^ that generating solutions scored by a design objective, here the length of the routes between elements $L(\vec{x}_i,\vec{x}_j)$, is described by the generating function

1 $$\begin{aligned} Z(\beta ) = \sum _{\{\vec{x}_i \in D\}} \sum _{\{R(\vec{x}_i,\vec{x}_j)\}} e^{-\beta \sum _{i,j} A_{ij} L(\vec{x}_i,\vec{x}_j)} \end{aligned}$$

where $\beta = 1/T$ is the inverse of the temperature (tolerance for non-minimal routing), and $R(\vec{x}_i,\vec{x}_j)$ is the set of possible connection routes between $\vec{x}_i$ and $\vec{x}_j$. $Z(\beta )$, which is known as a partition function in statistical mechanics, is a Laplace transform of the design objective that generates candidate arrangements at a frequency weighted by a pressure $\beta$ for minimal routing. I.e., at large $\beta$ (equivalent to $T\rightarrow 0$), Eq. 1 generates only arrangements with minimal or near-minimal routes, and generates routes of increasing *L* as $\beta \rightarrow 0$ (equivalent to $T\rightarrow \infty$).

The form of arrangements that *Z* generates is determined by how the multiplicity of options for routing a connection grows with the connection length. To see this, take the cardinality of the set of routes *R* between $\vec{x}_i$ and $\vec{x}_j$ as $\Omega _R(\vec{x}_i,\vec{x}_j)$ which gives the generating function as

2 $$\begin{aligned} Z(\beta ) = \sum _{\{\vec{x}_i \in D\}} e^{-\beta \sum _{i,j} A_{ij} \left( L(\vec{x}_i,\vec{x}_j)-\frac{1}{\beta } \ln \Omega _R(\vec{x}_i,\vec{x}_j)\right) } \end{aligned}$$

The factor in the exponent can be written as an effective distance

3 $$\begin{aligned} \Delta (\vec{x}_i,\vec{x}_j) = L(\vec{x}_i,\vec{x}_j)-T\ln \Omega _R(\vec{x}_i,\vec{x}_j) \end{aligned}$$

which expresses that routing multiplicity $\Omega _R$ counteracts the drive for minimal length *L* with a strength that is determined by the threshold for non-minimal routing *T*. Notably, $\ln \Omega _R$ is the Boltzmann entropy in statistical mechanics, the same physical property that drives clustering observed at the nanoscale^46,47^. This quantitatively connects known nanoscale clustering mechanisms to arrangement clustering. In routing problems where $\ln \Omega _R(\vec{x}_i,\vec{x}_j)$ grows sufficiently fast, e.g. combinatorially, there will be a threshold *T* that induces $\Delta (\vec{x}_i,\vec{x}_j) < 0$ via entropic repulsion.

In microscopic systems, entropic repulsion generates clustering at inhomogeneities either generated by symmetry breaking^46,47^ or at pre-existing inhomogeneities at boundaries^49^. Bounded, inhomogeneous domains, which induce sites of microscale clustering,^49^ are also generic in distributed systems. In our model system, it was precisely at the boundary where inhomogeneities clustering emerged, and this phenomenon should be generic.

Our analysis indicates that clustering occurs generically, and is driven by one of two mechanisms. Design elements can cluster to minimize connection distance, i.e. by minimizing $|\Delta |$ for $\Delta >0$ in Eq. 3. Or, elements can cluster emergently by entropic repulsion, i.e. by maximizing $|\Delta |$ for $\Delta <0$ in Eq. 3. Since clustering occurs for both $\Delta >0$ and $\Delta <0$, the only way to avoid clustering is if $\Delta \approx 0$ for separation distances that are larger than the characteristic $R_g$ of attraction-driven clusters and smaller than the separation distance of boundary inhomogeneities where repulsion-driven clusters localize. However, when $\Delta \approx 0$, the effects of minimal routing length and routing multiplicity counteract one another. In this regime, the system is driven by the configurational entropy of the arrangement of the elements, with the result being large variability in *L*. This means that avoiding clustering only occurs at the expense of high variability in the design objective.

### No free lunch and clustering

Clustering vulnerabilities present a serious challenge in a broad range of distributed systems engineering problems. We analyzed systems under minimal, information theoretic motivated assumptions. We argued that spatial features, which arise from physical considerations, and topological features, which arise from logical features, create an interplay integral to both the emergence of clustering and to the mitigation of the vulnerabilities clustering creates. We investigated the logical/physical interplay of clustering using an example from naval architecture for a trio of reasons; (1) naval architecture constitutes a setting where the physical/logical interplay emerges clearly; (2) naïve, “one-and-done” optimization approaches fail miserably for naval architecture problems because the timescales for development, fabrication, and deployment vastly outstrip the timescale for technological change; and (3) naval architecture clustering problems arise via driving factors that are mirrored in other domains (i.e., heterogeneity of connectivity and drivers for short physical connections) which suggested the outlines of the much more general argument we sketched above.

Based on the principle that since naïvely optimal design solutions generate clustering vulnerabilities for very generic reasons, mitigating clustering and its attendant vulnerabilities must be premised on a systematic analysis of solutions that are not strictly optimal. We analyzed non-optimal solutions, both in the naval architecture example and in the general case, by making use of a Pareto-Laplace filter^38^.

Our Pareto-Laplace filter based analysis showed that clustering vulnerabilities arise through both straightforward and unexpected, emergent mechanisms in distributed systems. It is possible that the clustering features found manifest differently with more space available in the geometry or with larger area, and the model can be tuned to reflect larger distance through the objective function. However, in the ship design problem, the spatial resolution used is sufficient for the early stage design shown in Refs.^14,50^. A small shift in scale will not affect the probabilistic behaviour of the distribution, and thus preserves the clustering behaviours. The clustering behaviour can also be different in systems with a much smaller number of routings $\ln \Omega _R(L)\lesssim L$, or systems that allow for more expensive, non-minimal routing paths. In addition, we used information theory to impose minimally-biased models of missing design information. It would be possible to modify the present approach by adding a bias that distinguishes between configurational and conformational entropy, i.e., by introducing a numerical coefficient that quantifies an “exchange” rate between those factors. Introducing such a factor, however requires additional information about potential design outcomes that may not be known *a priori*. Our results indicate that the variety of mechanisms that drive clustering echoes the familiar adage that “there is no free lunch” to eliminate the vulnerabilities that arise from clustering.

The naval architecture problem explored in this paper is one of many problems that deal with embedding fixed networks into a low-dimensional space with a fixed boundary such as problems in microprocessors and airlines^51,52^, where close spatial proximity of nodes leads to vulnerability. We show how the combinatorial space of possible routings necessarily gives rise to either attractive or repulsive clustering, or high design variability. With no *a priori* preference between those regimes, there is no problem-solving advantage at any specific value of *T*. Though the *existence* of spatial clusters is a necessity in low-*T* and high-*T* regimes, the *structure* of those clusters can be affected by the topological features of networks, such as broader degree distributions and assortative mixing.

**Figure 5:**
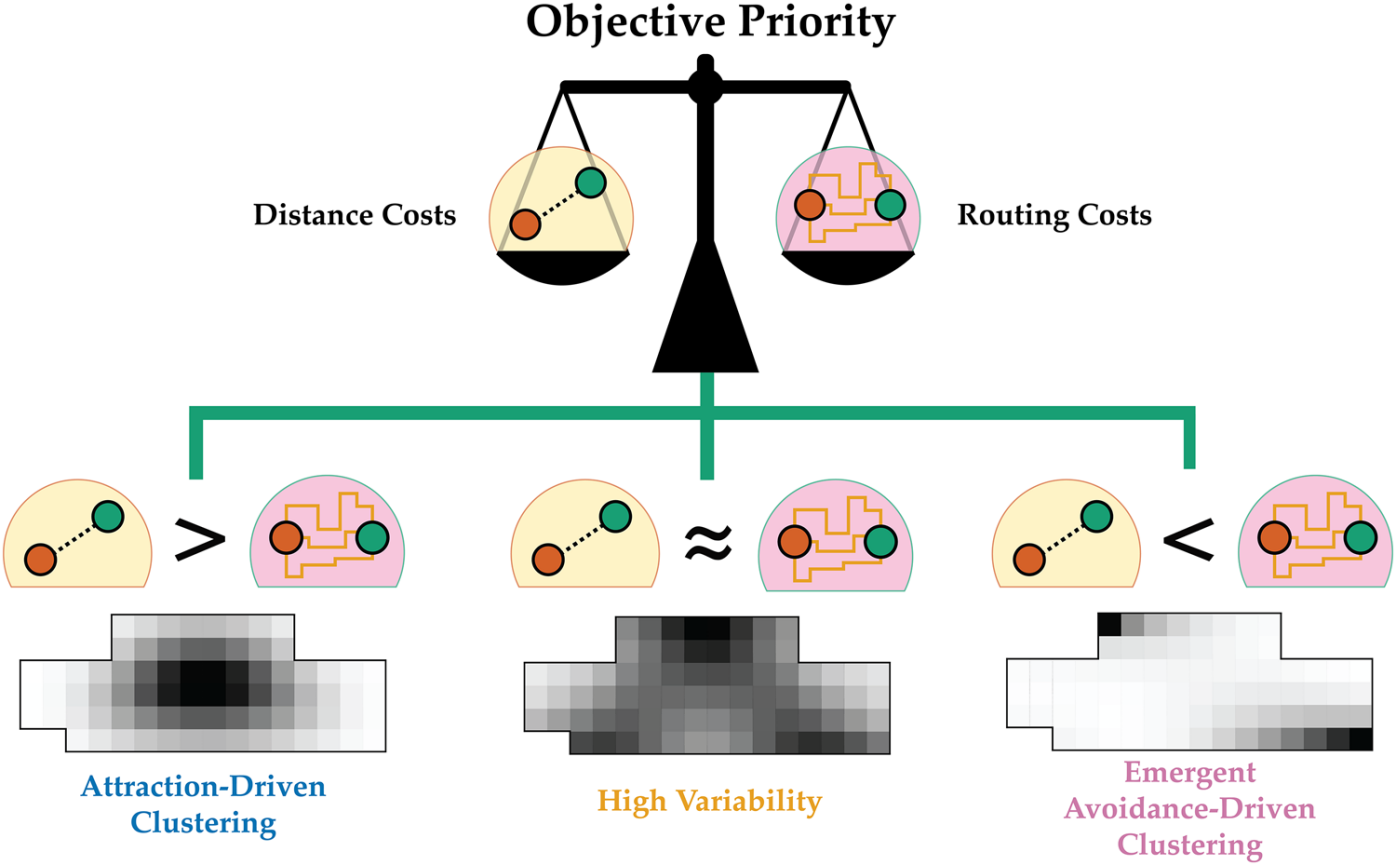
Trade-offs between multiple forms of clustering and objective variability imply that there is no free lunch to disrupt clustering. The attraction-driven clustering is an effect of the distance cost degrees of freedom, while the emergent avoidance-driven clustering is of the domination of the routing cost degrees of freedom. The configurations where the competing degrees of freedom are balanced produce high variability. Adopting high variability solutions circumvents the emergent clustering altogether.

We have shown that there is a complex mechanism involving the competing degrees of freedom and how element connectivity is arranged, akin to having configurational and conformational entropies. This competition among degrees of freedom drives emergent clustering. Furthermore, we have shown that clustering behaviour is dependent on the design-element connectivity. This complex mechanism can be avoided altogether by adopting high variability configurations. The trade offs in degrees of freedom shown in Fig. 5 further emphasize that there is always a cost associated with mitigation of clustering such as having high uncertainties of the design with high variability. Consequently, if clustering mitigation is a priority, designers can adopt high variability configurations by choosing not to precisely allocate elements to specific placements, but instead allocate rough placement regions. The inexact allocation can circumvent the emergent clustering mechanism. Precise allocation decisions should be made downstream where sources of clustering can be identified and managed. Although having low resolution in the early stage design is common, it may pose a challenge on early cost determination. Such is the price of the high variability strategy since there is “no free lunch” for avoiding clustering vulnerabilities.

## Methods

### Model

#### Considerations for model construction/selection

The clustering effects we observed in this paper depends on the close interplay of network topology and spatial constraints^37^, but the approach we take differs from approaches in other spatial network studies. Space-first studies usually first fix the spatial locations of the nodes and either study the empirical topology of the links or propose a distance-based model of link probability^7,53,54^. Depending on the model parameters, the resulting spatial networks can manifest different global topological features, such as the small-world effect, topological clustering, or assortative mixing by node degree^6,55,56^. In contrast, network-first studies start with a network topology and map nodes to coordinates into a high-dimensional Euclidean or hyperbolic space to study community structure, link prediction, and network navigability^57,58^. Though those approaches illuminate many spatio-topological features of large real-world networks, those approaches are not well suited to describe embedding a fixed network into a prescribed low-dimensional space with a fixed complex boundary.

Directly embedding networks into a low-dimensional space (2D or 3D) is important for physical realization. When both nodes and edges have finite size and cannot overlap, the resulting spatial embeddings show different regimes of complex structure and mechanical response. This phenomenon is mirrored in the evolved constraints on neuronal connections in mammalian brains induced by the space^59^. Similar considerations arise in network visualization on a 2D screen or page^60^. There, the visual structure of network layouts is often created through force-directed layout algorithms, and thus naturally highlights the community structure of the network^58^.

Real world networks can exhibit strongly disassortative and bipartite structure with connections only exist between low-degree and high-degree nodes. It is therefore instructive to consider example systems in this class.

#### Naval architecture example

For the naval architecture model system, we chose the power system elements and connectivity from Ref.^14^ shown in Panels A and B in Fig. 1. The power sources, MAIN and AUX, are the design elements with high connectivity. The low-connectivity elements are not logically connected to one another. The chosen network is a practical arrangement of possible connectivity configurations. With this connectivity, we construct a thermodynamic model using Systems Physics^39^.

For the design geometry of the model, we reproduce the geometry from Ref.^14^ with twice the resolution in each axis, resulting in more available spaces for design elements to occupied. The geometry is chosen such that it reflects a practical requirement in naval ship design from Ref.^14^.

To investigate the nature of clustering vulnerabilities, we created a computational method described in the following section to compute a vital statistical property known as partition function for the model distributed system. The model Naval Engineering system contains 16 design elements of which 14 elements are low connectivity (degree $k \le 2$) and 2 elements are high connectivity ($k > 2$). The logical connections between the elements are shown in panel (A) in Fig. 1. To obtain a design solution/configuration, the elements are placed in the ship geometry. An example of a design solution for the model distributed system is shown in panel (B) in Fig. 1.

### Solution space Pareto-Laplace filter

Formal approaches to design problems have been discussed previously, notably in Ref.^61^.

Here, we follow Ref.^14^ and consider a system with the objective function

4 $$\begin{aligned} \mathscr{O} = C \sum _{i,j} A_{ij} L(\vec{x}_i, \vec{x}_j). \end{aligned}$$

Here, *C* is a cost scaling constant, which we take as one to set units. *L* is the minimum Manhattan distance between two elements $\vec{x}_i$ and $\vec{x}_j$. $A_{ij}$ is an adjacency matrix where matrix elements for connected design elements are one, and matrix elements for unconnected design elements are zero. The nominal goal of the design problem is to minimize $\mathscr{O}$ via an appropriate placement of elements.

Ref.^38^ used geometric, information-theoretic, and physical arguments to argue that it is instructive to filter potential design solutions according to the value of the objective $\mathscr{O}$.

Instead of simply optimizing, Ref.^38^ proposes a Laplace transform of the space of possible realizations of the design problem by accumulating the set of possible design solutions that achieve some particular value of $\mathscr{O}$, according to

5 $$\begin{aligned} Z(\beta ) = \int d\mathscr{O} e^{-\beta \mathscr{O}} \Omega _\perp (\mathscr{O}), \end{aligned}$$

where $\beta$ is a Laplace transform variable, $\Omega _\perp (\mathscr{O})$ is the solution space volume at fixed objective. and is given by

6 $$\begin{aligned} \Omega _\perp (\mathscr{O}) = \sum _{\{\vec{x}_i \in D\}} \sum _{\{R(\vec{x}_i,\vec{x}_j)\}} \delta (\mathscr{O}-\mathscr{O}(\{\vec{x}_i\})), \end{aligned}$$

and where $R(\vec{x}_i,\vec{x}_j)$ is a convenience function for enumerating the minimal paths connecting design elements *i* and *j* given locations $\vec{x}_i$ and $\vec{x}_j$.

The filter in Eq. (5) has the effect of filtering out sub-optimal solutions depending on the magnitude of the parameter $\beta$. To understand the effect of the filter, it is simplest to make the change of variables $\beta =1/T$, from which it is possible to identify *Z* as a partition function in statistical mechanics where *T* is mathematically equivalent to temperature^38^. This gives

7 $$\begin{aligned} Z(T) = \sum _{\{\vec{x}_i \in D\}} \sum _{\{R(\vec{x}_i,\vec{x}_j)\}} e^{-\frac{1}{T}\mathscr{O}(\{x_i\})}, \end{aligned}$$

where we have absorbed the volume factor in Eq. (5) folding the solution space summation into the filter directly^38^.

Reference^38^ showed that *Z*(*T*) acts as a generating function for potential solutions in generic design problems. In the present context, the filter has the advantage that for $T\ll 1$, *Z* generates solutions that have small separation, *L*. In addition, since there are generically more ways of routing paths between elements with larger *L*, if $T\gg 1$, *Z* will generate solutions with more widely separated design elements. We compute the filter numerically using tensor networks following a method established in Ref.^37^ using open source code available at Ref.^62^.

With the requirements of the model system, we investigate the thermodynamic behaviour of each type of connectivity: high and low. The design elements that are logically connected should directly affect clustering since the objective function relies on the possible routing path and distance. We allow the system to vary the temperature, *T*, where the resources are controlled such that we can examine clustering at various conditions. At low *T*, the design elements are penalized by being at a distance to one another, since the allowed resources are lower. While at high *T*, although the resources are more available, the cost of routing can be a key factor in penalizing certain expensive configurations. Thus, the clustering vulnerabilities may emerge from temperature conditions at both high and low *T*.

### Critical temperature determination

In a system in the thermodynamic limit, one common indicator of a phase transition is the divergence of the isochoric heat capacity^43^. A phase transition indicates different regimes of thermodynamic behaviour, and hence, we utilize the temperature at which a phase transition occurs to define the boundary between two distinct regimes of behaviour. However, in a finite-sized system, the divergence manifests in the form of a maximum^63^. To determine the critical temperature $T_c$ in our system, we identify the temperature at which the heat capacity is maximum as shown in Fig. 3B.

### Connectivity-based clustering: radius of gyration

For the effects of clustering from connections from any pair of elements, we use the order parameter radius of gyration. The radius of gyration quantifies the likelihood of a pair of chosen elements to be close to one another, and is defined by:

8 $$\begin{aligned} R_g = {[\langle p(\vec{x}_i, \vec{x}_j)({(\Delta _{ij} x)}^2 + {(\Delta _{ij} y)}^2) \rangle ]}^{1/2} \end{aligned}$$

where $p(\vec{x}_i, \vec{x}_j)$ denotes the two-point correlation function between two units *i* and *j*, and the difference $\Delta$ is calculated as the distance between two points. We use an average, denoted by $\langle \cdots \rangle$, of correlation and distance between all possible pairs of elements to determine the radius of gyration. Hence, the radius of gyration represents an average clustering between all pairs of elements.

### Connectivity-agnostic clustering: design freedom

Measuring clustering without reference to element connectivity is akin to measuring how elements localize in a space. Emergent localization is a well-studied phenomenon in physics^64^, and we follow Ref.^37^ and use a normalized version of the existence area defined by

9 $$\begin{aligned} \Phi = \frac{1}{Y_0} \frac{{\bigg (\sum _{\vec{x}}p(\vec{x})\bigg )}^2}{\sum _{\vec{x}}{p(\vec{x})}^2} \end{aligned}$$

where the normalization $Y_0$ represents the number of available cells in the system, and $p(\vec{x})$ is a distribution of unit arrangements. Since probabilities are used instead of eigenmodes commonly found in the definition of existence area, the numerator evaluates to one. In this form, $\Phi$ is bounded above by one when $p(\vec{x})$ is uniform, and decreases monotonically to $1/Y_0$ as *p* becomes more localized. Because $\Phi$ describes the effective fraction of the total area free to unit placement, we refer to this as design freedom. The design freedom is calculated for each element, and in the context of a system, the design freedom is defined as an average over all elements.

### Design objective uncertainty

For the heat capacity, we find that it has a maximum at a temperature at which we define as a critical temperature of the model system. A divergence in heat capacity is an indicator of a phase transition in magnetic or other conventional thermodynamic systems^43^. The present system is finite-sized, so the heat capacity cannot diverge. A maximum in heat capacity indicates that there are distinct regimes of behaviour. In our case, the maximum in heat capacity suggests that there may be different behaviour in the temperature regimes separated by the maximum. Consequently, we define the temperature $T_c$ at which the maximum occurs as the critical temperature in the system. Panel B in Fig. 3 shows a finite maximum of heat capacity of the model system which we use to define the critical temperature.

### Supplementary Information

Supplementary Information 1.

## Data Availability

The datasets used and/or analysed during the current study available from the corresponding author on reasonable request.
