# Supplementary material for: No free lunch for avoiding clustering vulnerabilities in distributed systems
Source: Sci Rep. 2024 Jun 4;14:12789. doi: 10.1038/s41598-024-63278-3 (PMC11150256; doi:10.1038/s41598-024-63278-3)
Supplement: Supplementary file 1 — Supplementary Information 1. [file 41598_2024_63278_MOESM1_ESM.pdf]

Supplementary Information: No Free Lunch for Avoiding Clustering Vulnerabilities in Distributed Systems

Pheerawich Chitnelawong[1], Andrei A. Klishin[2,3], Norman Mackay[1], David J. Singer[4], and Greg van Anders[1,*]

[1]Department of Physics, Engineering Physics, and Astronomy, Queen's University, Kingston ON, K7L 3N6, Canada
[2]Department of Mechanical Engineering, University of Washington, Seattle, WA 98195, USA
[3]AI Institute in Dynamic Systems, University of Washington, Seattle, WA 98195, USA
[4]Department of Naval Architecture and Marine Engineering University of Michigan, Ann Arbor, MI 48109, USA
[*]gva@queensu.ca

ABSTRACT

Emergent design failures are ubiquitous in complex systems, and often arise when system elements cluster. Approaches to systematically reduce clustering could improve a design's resilience, but reducing clustering is difficult if it is driven by collective interactions among design elements. Here, we use techniques from statistical physics to identify mechanisms by which spatial clusters of design elements emerge in complex systems modelled by heterogeneous networks. We find that, in addition to naive, attraction-driven clustering, heterogeneous networks can exhibit emergent, repulsion-driven clustering. We draw quantitative connections between our results on a model system in naval engineering to entropy-driven phenomena in nanoscale self-assembly, and give a general argument that the clustering phenomena we observe should arise in many distributed systems. We identify circumstances under which generic design problems will exhibit trade-offs between clustering and uncertainty in design objectives, and we present a framework to identify and quantify trade-offs to manage clustering vulnerabilities.

Supplementary Text

Systems Physics Framework

Systems physics constructs a mathematical framework to study trade-offs in design problems using information theory formulation of statistical mechanics. This section follows the construction of *systems physics*[1]. Instead of the standard energy levels and external parameters of a mechanical system, we analyze how design objectives are affected by plausible set of design solutions. We introduce the *design objective* $\mathscr{O}(\alpha)$ where α is a *design solution* existing in a combinatorically large set of candidate designs $\{\alpha\}$. We substitute the design objective as the "energy level" function in the formulation. In a similar manner, we maximize entropy with the Lagrangian[1],

$$\mathscr{L} = -\sum_\alpha p_\alpha \ln p_\alpha - \sum_i \lambda_i \left(\sum_\alpha p_\alpha \mathscr{O}_i(\alpha) - \langle \mathscr{O}_i \rangle \right), \tag{S.1}$$

where λ_i are the Lagrange multipliers that force the objectives to match design outcomes. Entropy maximization then yields the normalized probability distribution

$$p(\alpha) = \frac{1}{Z} e^{-\sum_i \lambda_i \mathscr{O}_i(\alpha)}, \tag{S.2}$$

where Z is the partition function given by

$$Z = \sum_\alpha e^{-\sum_i \lambda_i \mathscr{O}_i(\alpha)}. \tag{S.3}$$

The Lagrange multipliers λ_i represent the *design pressures* which quantify the amount of effect an objective has on the system[2]. The choice of design pressure will determine the shape of the probability distribution, and hence determine the preferable design solutions.

In a design problem, the design pressure does not affect all the design components homogeneously. The effects of constraints depend on the connectivity of the components. Thus, the *effective design objective* $\mathscr{O}_{\text{eff}}$ is defined by

$$\lambda \mathscr{O}_{\text{eff}} = \sum_{i,j} A_{ij} f(\alpha), \tag{S.4}$$

where A_{ij} is an adjacency matrix representing the design component connectivity, and $f(\alpha)$ determines the magnitude of connection (coupling) between two components. Using the effective design objective, the partition function becomes

$$Z = \sum_{\alpha} e^{-\sum_{i,j} A_{ij} f(\alpha)}. \tag{S.5}$$

For consistency, the choice of $f(\alpha)$ will be made such that the function incorporates the constraint parameter, such as, the temperature T.

Tensor Network as a Graphical Language for Computing Correlation in Design

Thermodynamic quantities rely on computing the partition function. As shown previously, the partition function sums over all possible tractable configurations, and hence, requires a large amount of numerical computation. We solve this problem by formulating the summation in terms of tensor products. This construction is a generalization of the transfer matrix methods[3]. The logical connections between the design elements are encoded into the contraction indices between the tensors. The following sections will discuss how tensor network method is implemented for statistical mechanics in design problems[4].

Network Construction

Partition Function

To compute the partition function of a network, we use tensors as the computing "elements." The partition function in Equation S.5 can be written in a factorized form as[4]:

$$Z(\alpha) = \sum_{\alpha} \prod_{\substack{i<j: \\ A_{ij} \neq 0}} e^{-f(\alpha)}. \tag{S.6}$$

Each term in the summation is encoded in a *coupling tensor*. Figure S1 shows a graphical representation of a tensor network with the connectivity given by an example adjacency matrix

$$A_{ij} = \begin{pmatrix} 0 & 0 & 0 & 0 & 1 \\ 0 & 0 & 1 & 1 & 1 \\ 0 & 1 & 0 & 1 & 0 \\ 0 & 1 & 1 & 0 & 0 \\ 1 & 1 & 0 & 0 & 0 \end{pmatrix}. \tag{S.7}$$

Figure S1 gives an example of network diagram for a partition function from the connectivity defined by the adjacency matrix in Eq. S.7. The coupling tensors are depicted as yellow squares. The pink circles refer to the site tensors. A site tensor is an indicator tensor which is defined as the Kronecker delta ensuring that the connecting sites perceive the unit at the same location. For example, unit 1 site tensor has the Kronecker delta $\delta_{\alpha_{12}\alpha_{13}\alpha_{14}}$ since unit 1 is connecting the units 2, 3 and 4. The subscript α ensures that the summation is carried over all design solutions, in this case, all possible element placements. With site tensors, the partition for a network with n units is then given by

$$Z(\alpha) = \sum_{\alpha} \prod_{\substack{i<j: \\ A_{ij} \neq 0}} \delta_{(0)} \cdots \delta_{(n)} e^{-f(\alpha)}, \tag{S.8}$$

where $\delta_{(n)}$ represents the Kronecker delta of a unit n.

Probability Distribution

To calculate the probability density of where a component can be in the system, we attach an external degree of freedom into the site tensor representing the unit. In the graphical notation, we do this by adding an external leg to the site tensor, e.g., shown attached to unit 1 in Figure S2. In the example case, the contraction yields a rank-1 tensor (vector) representing the probability distribution, p, which can be normalized by dividing it by the partition function. The normalized distribution can be expressed as $\tilde{p} = p/Z$. Furthermore, two-point correlations can be computed by adding two external legs to the network in a similar manner.

Unit Anchor

In a network in which some design parameters are fixed, we encode a decision using an "anchor" site connecting to the corresponding unit. The anchor tensor is given by the Kronecker delta $\delta(\alpha, \tilde{\alpha})$ where $\tilde{\alpha}$ represents the design choice in the solution for the corresponding unit. Figure S3 shows an anchor site as green square attached to the site tensor of unit 1. In this case, unit 1 is fixed to a specific design choice given by the anchor tensor as the summation is executed.

Network implementation

A summation in a tensor network is called a *contraction*. One of the main challenges in contracting a complex tensor network is finding an optimal sequence at which the tensors are contracted. We use the computational package *TensorNetwork*[5] to handle the contraction sequence within the *Lachesis* package[6] that computes the thermodynamic quantities and correlations. *TensorNetwork* is mainly used in machine learning and strongly-coupled quantum systems. Our challenge is to create a network representing architectural designs which are vastly different from the standard usage of tensor networks. For efficiency, the data space of the study is managed by *Signac* data management[7–9].

Tree Tensor Decomposition

The challenging aspect of arrangement problem is that the design solution space is large. A large solution space results in large-dimension site tensors which require a large amount of computation memory for high-rank tensors. Since the site tensors are Kronecker deltas, we address this issue with *Tree Tensor* structure[10]. Tree tensor can be used to factorize a high-rank identity tensor into multiple interconnecting rank-3 identity tensors forming a *tensor tree*. Figure S4 shows the graphical representation of tree tensors for tensors from rank 4 to rank 8. Tensor tree decomposition reduces the memory cost significantly at the cost of network complexity. Tensor network contraction speed relies on how fast the algorithm can find an optimal contraction path. However, having more interconnecting tensors in the network may increase contraction significantly. Nevertheless, this compromise is necessary, because the memory required to store all the values in such large dimension tensor would render the network contraction unfeasible.

For example, the computational geometry from the model of this work has 78 points in space which translate to dimensions with 78 elements. A rank-3 tensor would require $78^3 = 474552$ elements to be stored. Consider a rank-6 tensor which occurs when there are six logical connections to a design unit, the number of elements to be stored is 2.25×10^{11} which may use over 225 GB of memory. When compared to an equivalent rank-6 tree tensor which contains $4 \times 78^3 = 1898208$ elements or approximately 2 MB of memory, the advantage of the tree tensor structure outweighs the network complexity cost. In our computation of the partition function, a single computation takes on average 0.18 seconds and requires approximately 135 MB of memory. The computation time scales with the size of the geometry and the complexity of connectivity since the tensor dimensions are dependent on the geometry size, and the tensor ranks are depend on the number of connections. The implementation of tree tensors reduces the memory usage at the cost of network complexity. Consequently, the network contraction time increases the more complex the network becomes.

References

1. Klishin, A. A., Shields, C. P., Singer, D. J. & van Anders, G. Statistical physics of design. *New J. Phys.* **20**, 103038, DOI: 10.1088/1367-2630/aae72a (2018). 1709.03388.
2. Klishin, A. A., Kirkley, A., Singer, D. J. & van Anders, G. Robust design from systems physics. *Sci. Reports* **10**, 14334, DOI: 10.1038/s41598-020-70980-5 (2020). 1805.02691.
3. Goldenfeld, N. *Lectures on Phase Transitions and the Renormalization Group* (Addison-Wesley, Reading MA, 1992).
4. Klishin, A. A., Singer, D. J. & van Anders, G. Avoidance, adjacency, and association in distributed system design. *J. Physics: Complex.* **2**, 025015, DOI: 10.1088/2632-072X/abe27f (2021). 2010.00141.
5. Roberts, C. *et al.* TensorNetwork: A library for physics and machine learning, DOI: 10.48550/ARXIV.1905.01330 (2019). 1905.01330.
6. Chitnelawong, P., Klishin, A. A., MacKay, N. & van Anders, G. Lachesis. Zenodo, DOI: 10.5281/ZENODO.8088164 (2023).
7. Adorf, C. S., Dodd, P. M., Ramasubramani, V. & Glotzer, S. C. Simple data and workflow management with the signac framework. *Comput. Mater. Sci.* **146**, 220–229, DOI: 10.1016/j.commatsci.2018.01.035 (2018).
8. Ramasubramani, V., Adorf, C. S., Dodd, P. M., Dice, B. D. & Sharon C. Glotzer. Signac: A Python framework for data and workflow management. In Akici, F., Lippa, D., Niederhut, D. & M. Pacer (eds.) *Proceedings of the 17th Python in Science Conference*, 152–159, DOI: 10.25080/Majora-4af1f417-016 (2018).
9. Adorf, C. S. *et al.* Glotzerlab/signac, DOI: 10.5281/zenodo.2581327 (2019).

10. Jermyn, A. S. Efficient tree decomposition of high-rank tensors. *J. Comput. Phys.* **377**, 142–154, DOI: 10.1016/j.jcp.2018.10.026 (2019).

11. Penrose, R. Applications of negative dimensional tensors. *Comb. Math. its Appl.* **1**, 221–244 (1971).

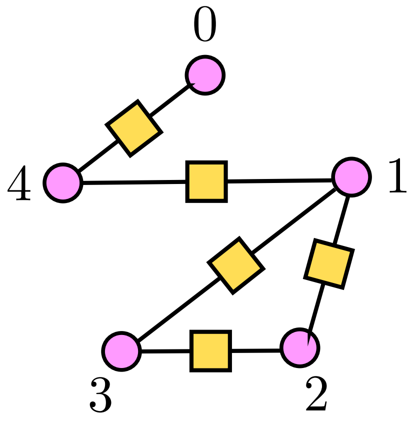

Figure S1. Partition function calculation is represented by a tensor network. A tensor network can be shown graphically with tensor notations by Penrose[11]. Each design element is embedded in the network as a node (pink circle). The connectivity between design elements is determined by the coupling tensors (yellow squares). The diagram shows an example of a tensor network with one mode of connectivity.

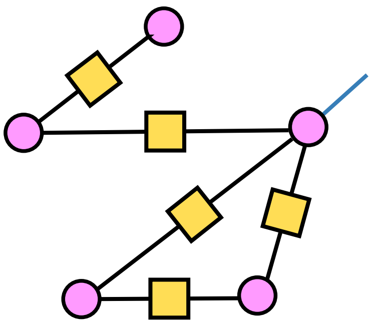

Figure S2. In the graphical tensor notation, to calculate a probability distribution for element placement, an external leg is introduced to the corresponding design element nodes. The diagram shows an example of graphical representation of a tensor network required to compute one-point function of unit one.

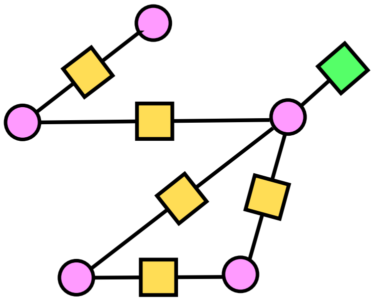

Figure S3. Partition function of the system with a fixed design element can be computed by connecting an anchor tensor to the corresponding design element node. The diagram shows a graphical representation of the partition function with fixed unit one.

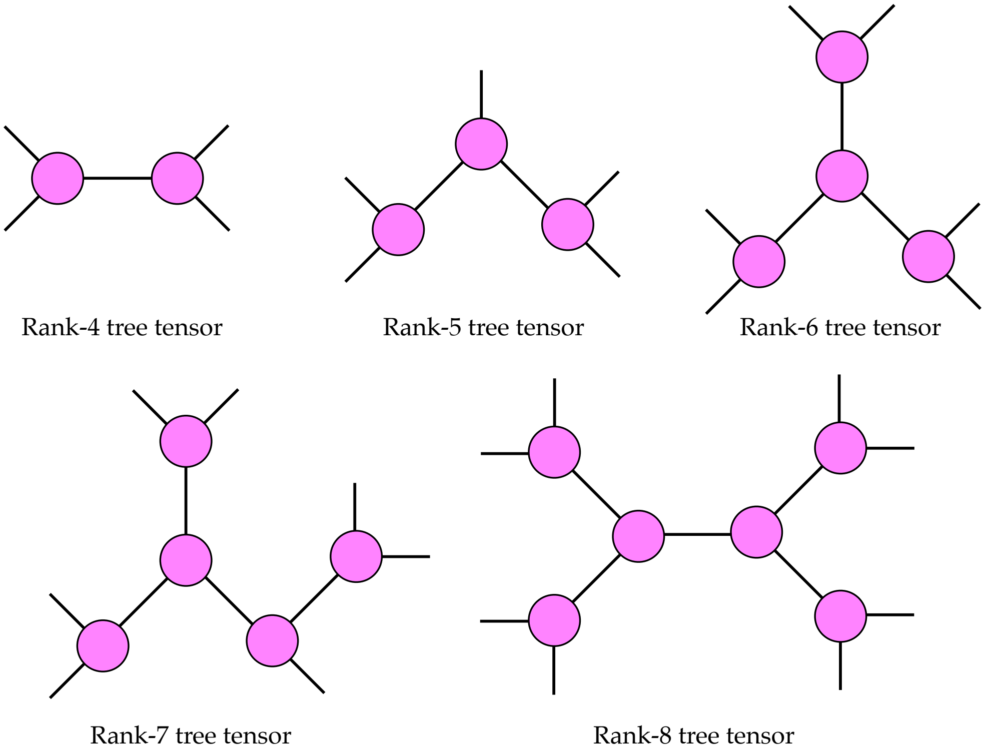

Figure S4. High rank tensors (rank three and above) decomposes into rank-3 tensors. In order to mitigate memory issues in computation, we decompose high rank tensors into a collection of tensors with tree tensor structure developed by Jermyn[10]. Diagram shows an example of high rank tensor decompositions for tensor of rank four to rank eight.
